# BMI can influence adult males’ and females’ airway hyperresponsiveness differently

**DOI:** 10.1186/2049-6958-7-45

**Published:** 2012-11-17

**Authors:** Bruno Sposato, Marco Scalese, Nicola Scichilone, Andrea Pammolli, Massimo Tosti Balducci, Maria Giovanna Migliorini, Raffaele Scala

**Affiliations:** 1 Unit of Pneumology, “Misericordia” Hospital, Via Senese 161, 58100, Grosseto, Italy; 2 Institute of Clinical Physiology, National Research Council (CNR), Pisa, Italy; 3 Section of Pneumology, Biomedical Department of Internal and Specialistic Medicine (DiBiMIS), University of Palermo, Palermo, Italy; 4 Department of Physiopathology, Experimental Medicine and Public Health University of Siena, Siena, Italy; 5 Unit of Nuclear Medicine, “Misericordia” Hospital, Grosseto, Italy; 6 Unit of Pneumology and UTIP, “S. Donato” Hospital, Arezzo, Italy

**Keywords:** Airway hyperresponsiveness, asthma, body mass index, males and females, methacholine test, obesity

## Abstract

**Background:**

Epidemiological data indicate that obesity is a risk factor for asthma, but scientific literature is still debating the association between changes in body mass index (BMI) and airway hyperresponsiveness (AHR).

**Methods:**

This study aimed at evaluating the influence of BMI on AHR, in outpatients with symptoms suggestive of asthma.

4,217 consecutive adult subjects (2,439 M; mean age: 38.2±14.9 yrs; median FEV_1_ % predicted: 100 [IQR:91.88-107.97] and FEV_1_/FVC % predicted: 85.77% [IQR:81.1-90.05]), performed a methacholine challenge test for suspected asthma. Subjects with PD_20_ < 200 or 200 < PD_20_ < 800 or PD_20_ > 800 were considered affected by severe, moderate or mild AHR, respectively.

**Results:**

A total of 2,520 subjects (60% of all cases) had a PD_20_ < 3,200 μg, with a median PD_20_ of 366 μg [IQR:168–1010.5]; 759, 997 and 764 patients were affected by mild, moderate and severe AHR, respectively. BMI was not associated with increasing AHR in males. On the contrary, obese females were at risk for AHR only when those with moderate AHR were considered (OR: 1.772 [1.250-2.512], p = 0.001). A significant reduction of FEV_1_/FVC for unit of BMI increase was found in moderate AHR, both in males (β = −0.255; p =0.023) and in females (β = −0.451; p =0.017).

**Conclusions:**

Our findings indicate that obesity influences AHR only in females with a moderate AHR level. This influence may be mediated by obesity-associated changes in baseline lung function.

## Background

A body of evidence has clearly demonstrated an association between obesity and asthma. In fact, obesity increases the risk of asthma and contributes to the occurrence of a more difficult-to-control phenotype of the disease [1]. Likely, this relationship is mediated by obesity-associated changes in lung function [1-3]. Indeed, the extra-pulmonary restrictive syndrome impairs the airway distensibility and causes a reduction in peripheral airway diameter which potentially increases airway hyperresponsiveness (AHR). In addition, hormonal and inflammatory factors, directly related to the adipose tissue, may influence some asthmatic phenotypes [1-3]. The adipose tissue expresses a number of pro-inflammatory molecules, such as leptin, tumour necrosis factor α (TNF-α), interleukin 6 (IL-6), transforming growth factor β1 (TGF- β1), adiponectin and C-reactive protein, which have been shown to modulate the airway inflammatory response thus promoting asthma [1-3].

To explain the mechanism underlying the relationship between obesity and asthma, the association between body mass index (BMI) and AHR has become the field of interest, leading, however, to conflicting results [1-3]. Several studies have shown a significant association between BMI and AHR [2,4-7], whereas others have failed to confirm this relationship [8-11]. An additional controversial issue is the different role of BMI with regard to gender. In some studies, specifically addressing the influence of gender, the relationship between BMI and asthma appeared to be significant only in females, whereas other investigations did not observe gender-associated differences [2,4,6,7,12,13]. The lack of definite data on the association between BMI and asthma may be due to several factors, such as the influence of asthma medications, the inclusion of relatively small numbers of patients and uncorrected asthma diagnosis. The use of inhaled corticosteroids, bronchodilators and anti-leukotriens can influence bronchial provocation test results [14,15]. Furthermore, when a higher cut-off value is used to define a positive bronchial provocation test, the asthma diagnosis may be overestimated [16] and this can obviously influence the outcome of the relationship with BMI.

To the best of our knowledge, there are no data specifically addressing the relationship between BMI and the different levels of bronchial hyperresponsiveness. With the assumption that there may be an association between BMI and asthma, we hypothesized that this should be different in the various AHR levels (i.e. mild, moderate and severe AHR). We therefore postulated that such a relationship should be more significant in subjects with a severe AHR rather than in subjects with a mild or moderate AHR.

Our retrospective study aimed at exploring in a large cohort of individuals whether there may be an association between BMI and AHR and if this association might be different in the various AHR levels. We also evaluated whether it may be affected by a possible relationship between BMI and baseline pulmonary function. In order to limit the influence of treatment, only subjects not taking asthma medications regularly were included in the study.

## Methods

### Subjects

A total of 4,217 consecutive adult subjects (2,439 M; mean age 38±14.9 yrs; median FEV_1_% predicted 100% [IQR: 91.88-107.97] and FEV_1_/FVC% 85.77% [IQR: 81.1-90.05]), who had performed a methacholine (Mch) bronchoprovocation test, were included. Subjects had been visited in the Pneumology Departments of Grosseto and Arezzo Hospitals (between 2000 and 2010) to confirm an asthma diagnosis. All subjects showed normal baseline lung function and had symptoms suggestive of asthma (unexplained episodes of cough and/or wheezing and/or dyspnea on exertion). FEV_1_, FEV_1_/FVC, FVC, FEF_25-75_ were obtained at baseline (pre-Mch test). The PD_20_FEV_1_ was calculated for each bronchoprovocation test and represented the main outcome of the study. Smoking habits, age, sex and BMI were also recorded for analysis. The use of data for the purpose of the study was approved by the local Ethic Committees.

None of the subjects was under regular treatment for asthma at the time of the test. Subjects, who had taken drugs when required, were asked to refrain from taking any medications prior to the Mch bronchoprovocation test. β_2_-agonist bronchodilators and inhaled or systemic corticosteroids were withheld 24 hours and 3 weeks before the test respectively, while antihistamines were stopped at least 10 days before the test. None of the subjects had suffered from airway infections or asthma exacerbations in the four weeks prior to the test. BMI was calculated by dividing the weight in kilograms by the square of the height in metres (kg/m^2^). According to the WHO classification, a BMI of < 18.5 kg/m^2^ describes a condition of underweight, 18.5-24.9 kg/m^2^ normal weight, 25–29.9 kg/m^2^ overweight and ≥ 30 kg/m^2^ obesity.

### Mch bronchoprovocation test

The Mch bronchoprovocation test was performed by using a dosimeter method [17]. The same instrument and method were used both in Grosseto and Arezzo in the years 2000–2010. Mch sulphate was supplied by Lofarma (Milan, Italy) and given in aerosol form using an MEFAR MB3 dosimeter (output: 9 μL/puff; MEFAR Elettromedicali Brescia, Italy) with an MB2 ampoule model. Buffer and Mch were diluted with distilled water and then two different progressive Mch solutions were obtained: an ampoule containing an Mch concentration of 4 mg/ml (40 μg inhalation dose) and another with 40 mg/ml (400 μg inhalation dose). The buffer solution was the first to be administered, followed by 40 μg of methacholine, increasing the doses until PD_20_FEV_1_ was obtained or until the maximum dose of Mch was reached. FEV_1_ was measured after inhaling 40, 80, 120, 240, 400, 800, 1,600, 2,400 and 3,200 μg of cumulative Mch doses, respectively. At the end of exhalation, during tidal breathing, patients inhaled Mch slowly and deeply from the nebulizer in 5 seconds and then held their breath for 5 additional seconds. The test was interrupted if a fall in FEV_1_>10% occurred with the buffer solution. The interval between two consecutive steps was 2 minutes. FEV_1_ was measured at 30 and 90 seconds after nebulization. An acceptable quality of FEV_1_ was achieved at each step. No more than two maneuvers after each dose were allowed and the highest FEV_1_ value was considered. AHR was defined by a 20% fall in FEV_1_ from the reference value (see below) obtained with a cumulative Mch dose < 3,200 μg. Subjects who did not achieve a 20% fall in FEV_1_ with a Mch dose of 3,200 μg were classified as normoreactive.

Subjects with PD_20_ ≤ 200, 200 < PD_20_ ≤ 800 and PD_20_ > 800 were arbitrarily considered as affected by severe, moderate and mild AHR respectively, with the purpose of evaluating the effects of BMI on the different levels of AHR.

Lung function was measured with an HP 47120E Pulmonary System Desk spirometer (Hewlett Packard, Waltham, Massachusetts - USA). At baseline, FEV_1_ and FVC were expressed as percentages of the predicted values, whereas FEV_1_/FVC was reported only as a ratio (reference equation: CECA, 1971). The PD_20_ FEV_1_ was assessed by linear interpolation of the dose–response curves. The FEV_1_ measured before administering the buffer solution was used as the baseline value, while the FEV_1_ measured after inhaling the buffer solution was used as reference value to calculate FEV_1_ decrease and thus PD_20_.

### Statistical analysis

Categorical variables were expressed as number of cases and percentages. Continuous variables were expressed as mean values and standard deviations or median values and interquartile range (IQR – 25° and 75° quartiles) according to whether they were normally distributed. Nonparametric or parametric tests were performed accordingly. Comparisons of qualitative and quantitative variables among groups were conducted by the chi-square test or the ANOVA one way test, respectively. Moreover, the Bonferroni test was used for multiple comparisons. The logistic binary regression model was applied separately in males and in females to evaluate independent risk factors for AHR. In order to evaluate the risk of the independent variables in the various levels of AHR (severe, moderate and mild), three logistic regression models were performed (both in males and females), considering subjects either with PD_20_>800 μg, or with PD_20_>200 and <800 μg, and with PD_20_<200 μg and comparing them with normal subjects. In these models, BMI was considered as qualitative variables, i.e. underweight, normal weight, overweight and obese subjects. The linear regression model was performed to evaluate whether a relationship existed between BMI and pulmonary function (FEV_1_, FVC, FEV_1_/FVC) in all degrees of AHR, adjusted for age, sex and smoking habits. P values < 0.05 were considered statistically significant. The statistical packages SPSS (16.0) was used for analysis.

## Results

**Figure 1:**
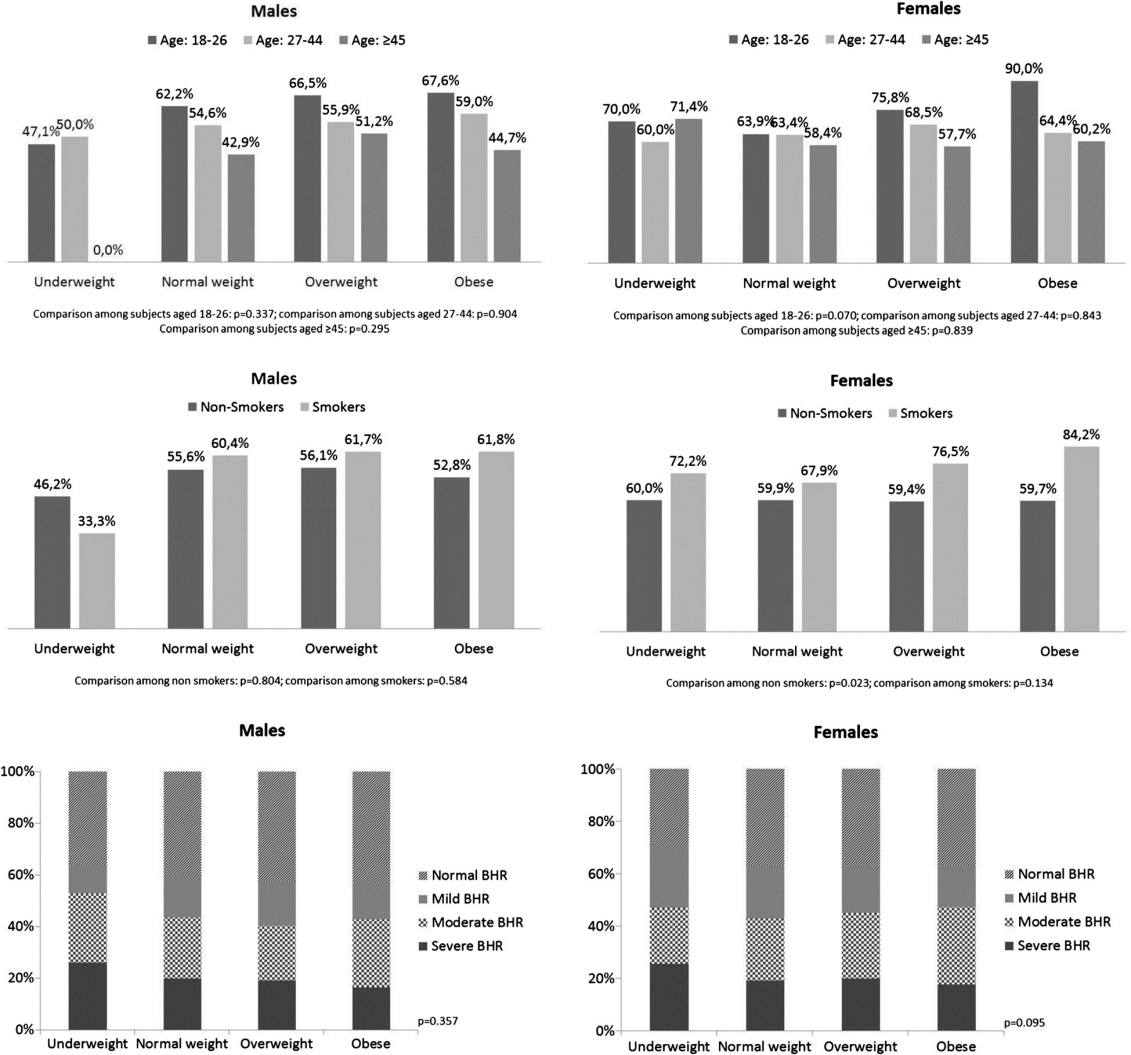
**Percentages of subjects with airways hyperresponsiveness defined by a PD_**20 **_< 3,200 μg in males and females according to weight, subdivided in the various categories taken into account.** Comparisons were made by using the *χ*^2^ test.

The subjects' characteristics are described in Table 1. FEV_1_ and FVC measured in underweight subjects were lower in comparison with those observed in normal weight, overweight and obese ones. FEV_1_/FVC was lower in overweight and obese patients if compared with normal weight individuals. On the contrary, FEV_1_/FVC was higher in underweight people. Hyperresponsive subjects (defined by a PD_20_< 3200 μg) were 2,520 (60% of the total), with a median PD_20_ of 366 μg [IQR: 168–1010.5]. In details, 759 had mild, 997 moderate and 764 severe AHR. Gender was equally distributed among the AHR groups. Both prevalence (Figure 1) and magnitude of AHR (Table 2 and Table 3) were similar among the different levels of BMI (underweight, normal weight, overweight and obese), in males and females and in different categories of subjects e.g. different ages, smokers, non-smokers and various AHR levels.

**Table 1:** Characteristics at baseline of 4,217 patients who underwent a methacholine challenge test

	**Underweight**	**Normal weight**	**Overweight**	**Obese**	**Total**	**p**
***N. patients (M/F)***	87 (20/67)*	2255 (1087/1168)*°	1300 (757/543)*° ^#^	575 (205/370)*° ^#^	4217 (2069/2148)	**0.0001**
***Age***	29±11*	34±13*°	42±15*° ^#^	47±15*° ^#^	38±15	**0.0001**
^***1***^***Current mokers (%)***	24 (31.2%)	490 (24.9%)	205 (17.6%)	72 (14.3%)	791 (21.3%)	
^***1***^***Ex smokers (%)***	1 (1.3%)	101 (5.1%)	127 (10.9%)	65 (12.9%)	294 (7.9%)	**0.0001**
^***1***^***Non-Smokers***	52 (67.5%)	1376 (70.0%)	831 (71.5%)	367 (72.8%)	2626 (70.8%)	
***Patients with normal reactivity***	34 (39.1%)	903 (40.0%)	530 (40.8%)	230 (40.0%)	1697 (40.2%)	0.969
***Patients with PD***_***20***_***<3200 μg***	53 (60.9%)	1352 (60.0%)	770 (59.2%)	345 (60.0%)	2520 (59.8%)	
***FEV***_***1***_***% of predicted***	96.96 [87.43-103.43]*° ^#^	100 [92.34-107.83]*	100 [91.79- 108.60]°	100 [90.26- 107.87]^#^	100 [91.88-107.97]	**0.007**
***FVC % of predicted***	92.06 [86.74-98.54]*° ^#^	98.84 [91.15-107.33]*	100.0 [92.16-108.36]°	99.77 [90.49-107.85]^#^	99.11 [91.21-107.60]	**0.0001**
***FEV***_***1***_***/FVC***	90.04 [84.94-95.03]*° ^#^	86.79 [81.80-91.56]*	84.69 [80.09-88.53]°	84.10 [80.76-88.20]^#^	85.77 [81.10-90.05]	**0.0001**
***BMI***	17.61±1.75*	22.17±1.71*°	27.10±1.39*° ^#^	33.53±3.90*° ^#^	25.14±4.58	**0.0001**

BMI, body mass index;FEV_1,_ forced expiratory volume in one second; FVC, forced vital capacity. The continuous variables are median (interquartile range [IQR]) or mean ± standard deviation and categorical values are expressed as number of cases (percentages). Mean comparisons were made by using the ANOVA test; median comparisons were made by using the Kruskal-Wallis test; proportion comparisons were made by using the *χ*^2^ test; *post-hoc* analysis was made by the Bonferroni correction.*° ^#^ statistically significant differences between groups when they were compared.

^1^valuated on 3,711 subjects whose smoking history was known.

**Table 2:** Median PD_**20 **_[IQR] values measured in males subdivided into various sub-groups taken into account

	**Underweight**	**Normal weight**	**Overweight**	**Obese**	**Total**	**p**
***Aged 18–26 years (n 556)***	656 [366–1589]	361 [169–852]	346 [166–846]	193 [146–356]	351 [168–848]	0.058
***Aged 27–44 years old (n 394)***	312 [312–312]	370 [150–981]	319 [165–1039]	354 [213–1032]	341 [161–1027]	0.871
***Age≥45 years (n 228)***	-	320 [104–1172]	360 [178–1133]	436 [277–903]	368 [180–1110]	0.343
***Severe AHR (PD***_***20***_***≤200) (n 367)***	117 [117–117]	95 [67–143]	115 [61–170]	116 [75–126]	105 [65–158]	0.159
***Moderate AHR (PD***_***20***_***>200 and ≤800) (n 471)***	377 [355–637]	377 [291–584]	351 [290–498]	353 [272–490]	357 [290–546]	0.294
***Mild AHR (PD***_***20***_***>800) (n 340)***	1855 [1322–3135]	1496 [1100–2097]	1530 [1105–2020]	1125 [1032–2167]	1437 [1094–2061]	0.142
***Non-smokers (n 794)***	507 [355–674]	343 [156–896]	343 [170–1036]	340 [192–825]	345 [168–944]	0.688
***Smokers (n 248)***	1589 [1322–1855]	547 [210–1060]	322 [169–1113]	352 [272–716]	442 [194–1072]	0.102
***Total (n 1178)***	637 [355–1322]	360 [156–928]	343 [169–1045]	355 [203–825]	353 [168–963]	0.555

AHR, Airways hyperresponsiveness. Comparisons were made by using the Kruskal-Wallis test.

**Table 3:** Median PD_**20 **_[IQR] values measured in females subdivided into various sub-groups taken into account

	**Underweight**	**Normal weight**	**Overweight**	**Obesity**	**Total**	**p**
***Aged 18–26 years (n 211)***	574 [75–1257]	371 [139–930]	330 [157–1491]	262 [137–663]	350 [140–1003]	0.763
***Aged 27–44 years (n 605)***	874 [213–1366]	455 [171–1101]	322 [159–697]	308 [155–917]	384 [165–1046]	0.065
***Age≥45 years (n 526)***	115 [96–373]	563 [197–1238]	384 [170–1063]	366 [205–848]	437 [179–1072]	0.160
***Severe AHR (PD***_***20***_***≤200) (n 400)***	59 [40–96]	94 [56–146]	107 [52–157]	105 [52–156]	98 [54–149]	0.125
***Moderate AHR (PD***_***20***_***>200 and ≤800) (n 523)***	369 [317–568]	437 [298–605]	363 [287–562]	355 [295–528]	383 [295–576]	0.858
***Mild AHR (PD***_***20***_***>800) (n 419)***	1366 [1176–1805]	1447 [1072–2026]	1484 [1097–2112]	1473 [1203–1948]	1451 [1097–2015]	0.064
***Non-smokers (n 889)***	468 [99–1169]	473 [157–1109]	382 [165–1077]	359 [180–814]	396 [161–1072]	0.765
***Smokers (n 273)***	1132 [72–1521]	502 [172–986]	332 [151–647]	321 [203–818]	407 [160–980]	0.449
***Total (n 1342)***	568 [108–1271]	490 [161–1077]	354 [160–964]	352 [179–817]	387 [165–1052]	0.359

AHR, Airways hyperresponsiveness. Comparisons were made by using the Kruskal-Wallis test.

We therefore assessed whether BMI was associated with the AHR level (Spearman correlation), as expressed by the PD_20_, in specific subgroups (Table 4), in females, a significantly negative correlation between BMI and AHR resulted in all hyperresponsive subjects aged 27–44 years (r = −0.113). This relationship was likely caused by the significantly negative association detected in females with moderate AHR (r = −0.210). A similar relationship was detected in younger (r = −0.257) and non-smokers (r = −0.116) females with moderate AHR. Interestingly, a highly positive significant correlation between BMI and PD_20_ was observed in underweight females with moderate AHR (r = 0.587), which probably influences the positive significant association in the entire group of underweight females.

**Table 4:** Spearman and partial correlation (r) between PD_**20 **_and BMI in males and females with different AHR levels divided into various sub-groups

	**All males with AHR**	**All females with AHR**	**Males with severe AHR**	**Females with severe AHR**	**Males with moderate AHR**	**Females with moderate AHR**	**Males with mild AHR**	**Females with mild AHR**
***All subjects with AHR (n. 2520)***	−0.003	−0.033	0.105*	0.067	−0.086	−0.115	−0.041	0.029
***Aged 18–26 years (n. 767)***	−0.038	−0.072	0.074	0.229	−0.066	−0.257*	−0.113	0.030
***Aged 27–44 years (n. 999)***	−0.031	−0.113°	0.062	−0.082	−0.135	−0.210^#^	−0.133	−0.088
***Age≥45 years (n. 754)***	0.092	−0.001	0.268*	0.076	0.092	−0.091	0.067	0.025
***Non-smokers (n. 1683)***	0.016	−0.012	0.101	0.069	−0.032	−0.116*	−0.084	−0.016
***Smokers (n. 521)***	−0.082	−0.057	0.185	0.216*	−0.239*	−0.111	0.099	0.048
***Underweight (n. 53)***	−0.30	0.288	-	−0.115	−0.100	0.587*	−0.500	−0.248
***Normal weight (n. 1352)***	0.011	−0.004	−0.024	−0.011	0.030	−0.061	−0.049	−0.016
***Overweight (n. 770)***	0.015	0.120*	0.038	0.070	−0.013	0.115	0.102	0.021
***Obese (n. 345)***	−0.065	0.098	0.018	−0.177	0.087	−0.097	−0.154	−0.044
***All subjects with AHR***^***1***^***(n 2198)***	−0.060	−0.050	0.104	0.042	−0.088	−0.161^#^	−0.113*	−0.032

Subjects with AHR = PD_20_ < 3200 μg; Severe AHR = PD_20_ ≤ 200 μg; moderate AHR = PD_20_ > 200 and ≤ 800 μg; mild AHR = PD_20_ > 800 μg; *p < 0.05; °p < 0.01; ^#^p < 0.001; ^1^correct for age, smoking habits, FEV_1_ and FVC, calculated on subjects where the smoking history was known.

In males, a strong inverse correlation was observed in the mild underweight group between BMI and PD_20_ (r = −0.500). A similar significant relationship was seen in smokers with moderate AHR (r = −0.239). Positive associations between BMI and PD_20_ were observed in individuals with severe AHR (r = 0.105) and in particular in those aged over 45 years (r = 0.268). When a partial correlation (adjusted for age, FEV_1_, FVC and smoking habits) was applied, significantly negative relationships between BMI and PD_20_ were confirmed in females with moderate AHR and males with mild AHR (Table 4).

The logistic regression model showed that in hyperreactive males the underweight condition appeared to be the protective factor against AHR (Table 5), although the relationship was weak (p = 0.047). In females, BMI was held as a risk factor for AHR only in obese subjects with moderate AHR (OR: 1.772 [1.250-2.512], p = 0.001) (Table 6). Age acted as a protective factor for AHR in all males (Table 5) and only in females (Table 6) with moderate and severe AHR groups. Smoking habit was a significant risk factor in females for all different levels of AHR, but not in males. FEV_1_ and FVC were risk and protective factors respectively for AHR both in males and females in all AHR levels.

**Table 5:** Logistic binary regression model to evaluate the AHR risk of the various considered covariates in males with different levels of airways hyperresponsiveness (compared to normal subjects)

	**All AHR subjects**^**a**^			**Mild AHR**^**a**^			**Moderate AHR**^**a**^			**Severe AHR**^**a**^		
	**OR**	**95% CI**	**p**	**OR**	**95% CI**	**p**	**OR**	**95% CI**	**p**	**OR**	**95% CI**	**P**
***Aged 27–44 years)***^***b***^	0.627	0.496-0.793	0.0001	0.757	0.545-1.051	0.097	0.605	0.450-0.813	0.001	0.572	0.407-0.804	0.001
***Age ≥ 45 years***^***b***^	0.453	0.344-0.596	0.0001	0.672	0.461-0.979	0.038	0.359	0.247-0.523	0.0001	0.408	0.272-0.610	0.0001
***Smoking***^***c***^	1.101	0.869-1.395	0.424	1.212	0.877-1.675	0.244	1.147	0.854-1.540	0.361	0.874	0.613-1.245	0.455
***FEV***_***1***_***% of predicted***	0.934	0.921-0.948	0.0001	0.969	0.950-0.989	0.002	0.936	0.919-0.954	0.0001	0.899	0.880-0.918	0.0001
***FVC % of predicted***	1.039	1.025-1.054	0.0001	1.009	0.990-1.029	0.360	1.043	1.025-1.062	0.0001	1.066	1.045-1.087	0.0001
***Winter***^***d***^	1.104	0.841-1.448	0.477	1.253	0.860-1.824	0.240	0.988	0.695-1.404	0.945	1.185	0.800-1.757	0.398
***Spring***^***d***^	1.103	0.849-1.434	0.463	1.106	0.765-1.599	0.592	1.157	0.829-1.614	0.392	1.229	0.840-1.799	0.289
***Summer***^***d***^	0.886	0.665-1.181	0.410	0.932	0.620-1.399	0.733	0.979	0.680-1.411	0.910	0.793	0.515-1.223	0.295
***Underweight***^***ef***^	0.384	0.149-0.988	0.047	0.466	0.125-1.734	0.255	0.596	0.198-1.791	0.357	-	-	-
***Overweight***^***ef***^	1.247	0.999-1.557	0.051	1.200	0.883-1.632	0.244	1.099	0.823-1.467	0.522	1.366	0.993-1.879	0.055
***Obese***^***ef***^	1.059	0.748-1.501	0.746	0.924	0.559-1.527	0.757	1.435	0.928-2.220	0.105	0.885	0.520-1.508	0.654

Subjects with AHR = PD_20_ < 3200 μg; Severe AHR = PD_20_ ≤ 200 μg; moderate AHR = PD_20_ > 200 and ≤ 800 μg; mild AHR = PD_20_ > 800 μg; ^a^vs subjects with normal reactivity (without PD_20_); ^b^vs age ≤ 26 years; ^c^vs non-smoking; ^d^vs Autumn; ^e^vs normal weight subjects; ^f^adjusted for age, smoking, FEV_1_, FVC and seasons.

**Table 6:** Logistic binary regression model to evaluate the risk of the various considered covariates in females with different levels of airways hyperresponsiveness (compared to normal subjects)

	**All AHR subjects**^**a**^			**Mild AHR**^**a**^			**Moderate AHR**^**a**^			**Severe AHR**^**a**^		
	**OR**	**95% CI**	**p**	**OR**	**95% CI**	**p**	**OR**	**95% CI**	**P**	**OR**	**95% CI**	**p**
***Aged 27–44 years***^***b***^	0.775	0.569-1.057	0.107	0.899	0.600-1.346	0.604	0.753	0.506-1.121	0.163	0.611	0.398-0.939	0.025
***Age ≥ 45 years***^***b***^	0.589	0.423-0.819	0.002	0.766	0.496-1.181	0.227	0.558	0.364-0.854	0.007	0.406	0.253-0.651	0.0001
***Smoking***^***c***^	1.609	1.243-2.081	0.0001	1.414	1.014-1.973	0.041	1.750	1.278-2.397	0.0001	1.767	1.229-2.540	0.002
***FEV***_***1***_***% of predicted***	0.925	0.912-0.939	0.0001	0.957	0.938-0.976	0.0001	0.928	0.910-0.946	0.0001	0.873	0.853-0.894	0.0001
***FVC % of predicted***	1.055	1.039-1.070	0.0001	1.029	1.009-1.049	0.003	1.061	1.041-1.080	0.0001	1.085	1.062-1.109	0.0001
***Winter***^***d***^	0.645	0.491-0.847	0.002	0.613	0.431-0.872	0.007	0.674	0.478-0.950	0.024	0.662	0.447-0.981	0.040
***Spring***^***d***^	0.717	0.541-0.949	0.020	0.585	0.403-0.849	0.005	0.810	0.572-1.147	0.235	0.829	0.557-1.236	0.358
***Summer***^***d***^	0.624	0.469-0.830	0.001	0.681	0.476-0.976	0.036	0.662	0.462-0.949	0.025	0.524	0.340-0.807	0.003
***Underweight***^***ef***^	1.012	0.568-1.805	0.967	1.222	0.611-2.443	0.571	0.774	0.340-1.766	0.543	0.895	0.390-2.055	0.793
***Overweight***^***ef***^	1.178	0.919-1.509	0.195	1.042	0.752-1.444	0.805	1.297	0.947-1.777	0.104	1.338	0.929-1.928	0.118
***Obese***^***ef***^	1.247	0.937-1.661	0.130	0.856	0.578-1.268	0.439	1.741	1.226-2.471	0.002	1.235	0.806-1.894	0.333

Subjects with AHR = PD_20_ < 3200 μg; Severe AHR = PD_20_ ≤ 200 μg; moderate AHR = PD_20_ > 200 and ≤ 800 μg; mild AHR = PD_20_ > 800 μg; ^a^vs subjects with normal reactivity (without PD_20_); ^b^vs age ≤ 26 years; ^c^vs non-smoking; ^d^vs Autumn; ^e^vs normal weight subjects; ^f^adjusted for age, smoking, FEV_1_, FVC and seasons.

In the attempt to find also a possible relationship between BMI and pulmonary function, we applied a regression linear model associating BMI and baseline lung function, adjusted for age, season and smoking habit (Table 7). Both in males and females, a relationship was found between BMI and FEV_1_/FVC in the entire group and in subjects with moderate AHR. In the latter, a significant reduction of FEV_1_/FVC for unit of BMI increase was found both in males (β = −0.255; p = 0.023) and females (β = −0.451; p = 0.017). No significant relationships between BMI and FEV_1_/FVC were found, either in males or females, both with severe AHR and normal bronchial response to Mch.

**Table 7:** Relations between lung function and BMI (regression linear model) corrected for age, season and smoking habits

	**All AHR subjects**				**Mild AHR**				**Moderate AHR**				**Severe AHR**				**Normal**			
	**Males**		**Females**		**Males**		**Females**		**Males**		**Females**		**Males**		**Females**		**Males**		**Females**	
	**β**	**p**	**β**	**p**	**β**	**p**	**β**	**p**		**p**	**β**	**p**	**β**	**p**	**β**	**p**	**β**	**p**	**β**	**p**
***FVC%***	−0.062	0.110	−0.155	0.004	−0.091	0.200	−0.071	0.437	−0.229	0.022	−0.388	0.011	−0.006	0.938	−0.102	0.537	−0.027	0.712	−0.183	0.037
***FEV***_***1***_***%***	0.064	0.092	0.132	0.015	0.069	0.330	0.037	0.689	0.228	0.021	0.371	0.014	0.028	0.717	0.100	0.537	0.027	0.712	0.152	0.081
***FEV***_***1***_***/FVC***	−0.118	0.009	−0.173	0.01	−0.177	0.049	−0.094	0.416	−0.255	0.023	−0.451	0.017	−0.044	0.623	−0.066	0.733	−0.105	0.220	−0.206	0.062
***Age***	0.075	0.0001	0.122	0.0001	0.051	0.012	0.140	0.0001	0.098	0.0001	0.090	0.0001	0.065	0.002	0.114	0.0001	0.074	0.0001	0.128	0.0001
***Seasons***	0.042	0.551	0.050	0.613	0.229	0.167	−0.131	0.566	−0.037	0.820	0.294	0.155	−0.001	0.996	−0.277	0.193	0.032	0.757	0.100	0.541
***Smoking***	−0.345	0.074	−0.917	0.001	−0.743	0.093	−1.251	0.054	−0.055	0.893	−0.835	0.139	−0.516	0.289	−0.656	0.267	−0.338	0.248	−1.147	0.027
***Constant***	32.22	0.0001	37.27	0.0001	40.00	0.0001	31.40	0.004	43.61	0.0001	62.22	0.0001	24.65	0.002	27.30	0.120	30.47	0.0001	40.32	0.0001

Subjects with AHR = PD_20_ < 3200 μg; Severe AHR = PD_20_ ≤ 200 μg; moderate AHR = PD_20_ > 200 and ≤ 800 μg; mild AHR = PD_20_ > 800 μg. Normal = subjects with normal reactivity (without PD_20_).

## Discussion

The current study was performed with the aim of exploring the relationship between BMI and AHR in a large cohort of male and female adults with suspected asthma. Overall, the proportion of hyperresponsive subjects did not differ with body weight in the population tested. Similarly, the level of AHR was equally distributed among the body weight groups. However, an interesting bi-modal behaviour emerged in females with moderate degree of AHR: a “protective” effect of body weight in the underweight condition and a “detrimental” effect of body weight in the obese state, thus describing an imaginary inverted U-shape curve of the relationship between BMI and PD20. These findings suggest that the influence of the adipose tissue on airway function should be further investigated in the moderate hyperresponsive female phenotype.

Our results are in line with some studies [18,19] that did not find any difference in PD_20_ values in obese and normal-weight asthmatics, and differ from those observed in the study of Schachter et al. [8], who found an increase in AHR in underweight subjects, although no plausible explanations were provided. Our results in the underweight subgroup are conflicting. In fact, in underweight females with moderate AHR, a significant positive correlation was found between BMI and PD_20_ which allowed us to infer that increasing weight might reduce the degree of AHR in these subjects. Therefore, the underweight condition in females seems to be a risk factor for AHR even though the logistic regression model did not confirm such a risk. On the contrary, the underweight condition in males seems to be a protective factor for AHR, probably due to hormonal differences.

In addition, our study showed that BMI (adjusted for age, pulmonary function, smoking and seasons) was an AHR risk factor in females but not in males. In fact, the obese status in females was an important AHR risk factor only when subjects with moderate levels of AHR were considered. Like other investigators, we could not find the expected relationship between BMI and PD_20_ in subjects with severe AHR, [20]. In fact, they found that obesity in asthmatic patients is negatively correlated to the intensity of AHR and not to asthma severity. This could be explained by the fact that in most severe stages, AHR is mainly characterized by a non-reversible component, probably associated with airway structural changes.

The observed different behaviours between sexes can already be found in literature. In fact, other studies showed differences between sexes with regard to the influence of BMI on AHR [2,4,6,7,12,13], with a more pronounced association in females [2,13,21,22]. It is plausible that sexual hormones, and in particular estrogens, may play a role in modulating the relationship between BMI and asthma. In obesity, the production of estrogens is generally increased and is associated with early menarche in women and delay in the onset of puberty in men. Some authors demonstrated that the prevalence of asthma, the association between BMI and the severity of the disease were greater in women with early menarche [23,24]. Furthermore, estrogens and progesterone may modify the inflammatory response to favour a Th2 response [1]. In this respect, β-estradiol enhances eosinophil adhesion to human mucosal microvascular endothelial cells and induces degranulation (unlike the testosterone effect), whereas progesterone increases bronchial eosinophilia and enhances bronchial responsiveness [25,26]. Another explanation may lie in the different abdominal fat distribution in males and females. In the latter, there is a greater subcutaneous fat distribution, whereas in males a higher visceral adipose tissue is observed. Interestingly, the subcutaneous abdominal fat appears to increase the risk of hyperresponsiveness, whereas visceral abdominal fat is not associated with AHR [27]. Furthermore, gynoid fat mass was associated with higher degrees of hyperrresponsiveness after an hypertonic saline challenge test in females [13]. Likely, the higher leptin production from subcutaneous fat rather than visceral fat, with greater values in females compared with males, may be the cause of a more serious status of asthma in obese women [1,28]. Leptin may activate or increase the airway inflammation in asthmatics [1,2,12,29]; in fact, a relationship between circulating leptin levels and risk of asthma development was observed in females [29]. Recently, an increase in neutrophilic airways inflammation in obese female asthmatics was documented in a study [12]. Another study showed that a gynoid fat mass is associated with a lower concentration of airway eosinophils in females [13]. Therefore, the different influence of BMI on AHR could be due to a different inflammatory pattern induced by obesity in males and females. Likely, a higher production of leptin from subcutaneous adipose tissue, which is typical of females, promotes T-helper type 1 cell differentiation and increases activation of neutrophils via tumour necrosis factor α [30].

The alternative explanation for our findings could be a pure mechanical factor, as demonstrated by the influence of the reduction in the FEV_1_/FVC ratio for unit of BMI increase (obstructive pattern). An excess soft tissue weight compressing the thoracic cage, a fatty infiltration of chest wall and an increase in pulmonary blood volume, might contribute to determine a reduction in lung volumes for a mechanical effect especially in females [2,20,31]. This is associated with an impairment in the lung inflation-induced airway distensibility and a reduction in airway peripheral diameter, which, over time, alter smooth muscle function thus increasing both airway obstruction and consequently AHR [1]. In our study, the FEV_1_ increase was a protective factor for AHR in both sexes, therefore suggesting that a reduction of lung function may favour AHR. It is likely that the already smaller calibre of airways in females may be influenced by BMI-induced obstruction in a more pronounced fashion than in males. This is supported by other studies who also observed a greater effect of adipose tissue on females’ lung function compared with males’ [13,32].

Another interesting result of this study, as already pointed out, is the different relationship between BMI and AHR at various levels of AHR (mild, moderate and severe) in females. The absence of any associations between BMI and mild AHR is certainly due to the fact that a great proportion of subjects belonging to this group did not result asthmatics. In fact, high values of PD_20_ or PC_20_, in case of suspected asthma (as in our patients), make an asthma diagnosis less probable [16]. It is difficult to explain why BMI and AHR are associated only in moderate hyperresponsive females and not in severe AHR. The factors that contribute to AHR may be divided into two categories: persistent and variable [33]. The airway structural changes (i.e. sub-endothelial and sub-basement membrane thickening, smooth muscle hypertrophy, matrix deposition, and altered vascular components - due to the chronic and long standing airway inflammation) represent persistent alterations [33]. On the other hand, the variable AHR component is believed to relate to inflammatory airway events, which may vary and be influenced by numerous environmental events (ie, allergens, respiratory infections and treatment) [16]. It is logical to hypothesize that these two components are interrelated. Obesity is a chronic inflammatory state [1,2] and may have a variable component role in the bronchial hyperreactivity mechanism. In fact, neutrophilic airways inflammation is increased by obesity and fatty acids in asthma [12]. Similarly, weight loss, through bariatric surgery, produces significant reductions in exhaled nitric oxide concentrations in obese asthmatic patients [34]. Proinflammatory molecules, expressed by adipose tissue such as leptin, TNF-α, IL-6, TGF- β1, adiponectin and C-reactive protein, increase in obese subjects [1,2,12], especially in females (above all C-reactive protein and leptin) [13]. These obesity factors may interfere with persistent AHR mechanisms and they may be greater in severe than in moderate AHR. In fact, the exhaled nitric oxide level increases significantly with the increasing of the AHR level in asthmatics [35,36]. Patients with intermittent asthma also showed airway inflammation but to a lower extent than those with persistent asthma [37]. Therefore, the supposed additional effects of inflammatory components, due to obesity (variable component), may have a lower impact in subjects with severe AHR because they have already a basal high level of asthma induced-inflammation (persistent component). On the other hand, basal airway inflammation may be less extensive in subjects with moderate AHR and therefore the variable inflammatory effect of weight may be greater, thus influencing AHR. In other words, systemic inflammation (BMI-induced) might influence airways inflammation only in asthma mild forms, whereas this influence might be trivial in more severe asthmatics. The fact that we could not find any relationships between BMI and pulmonary function in females with severe AHR strengthens our hypothesis.

Obesity may influence AHR in females with moderate hyperresponsiveness through a greater bronchial obstruction (reduced FEV_1_/FVC for unit of BMI increase). Reduction in FEV_1_/FVC was also found both in obese children [7] and adults [20], but not confirmed by Scott et al. [13]. This low level of FEV_1_/FVC may be a consequence of a systemic inflammation induced by adipose tissues or simply the mechanical effect of weight. When AHR becomes severe, the relationship between BMI and pulmonary function disappears so that we may hypothesize that the factors responsible for AHR may be only due to airway inflammation but probably not to systemic BMI induced inflammation and probably not to mechanical induced obstruction either.

## Conclusions

In conclusion, obesity can influence airway hyperresponsiveness only in females and only when the AHR level is moderate. No effects of BMI were found in males and in severe AHR. This BMI-induced influence may be mediated by a greater airway obstruction, probably due to a systemic inflammation or a purely weight associated mechanical effect.

## Competing interests

All authors declare to have no conflicts of interest, including specific financial interests and relationships and affiliations relevant to the subject of the manuscript.

## Authors’ contribution

All the authors certify that the material is original and not being considered for publication elsewhere. The authors alone are responsible for the contents and writing of the paper. All authors have given a significant contribution and have read and approved the submission of the manuscript.

## References

[B1] Beuther DA Weiss ST Sutherland ER Obesity and asthma Am J Respir Crit Care Med 2006 174 112 119 10.1164/rccm.200602-231PP 16627866 PMC2662903

[B2] Sharma S Tailor A Warrington R Cheang M Is obesity associated with an increased risk for airway hyperresponsiveness and development of asthma? Allergy Asthma Clin Immunol 2008 4 51 58 10.1186/1710-1492-4-2-51 20525125 PMC2868882

[B3] Shore SA Obesity, airway hyperresponsiveness, and inflammation J Appl Physiol 2010 108 735 743 10.1152/japplphysiol.00749.2009 19875711 PMC2838631

[B4] Chinn S Jarvis D Burney P European Community Respiratory Health Survey: Relation of bronchial responsiveness to body mass index in the ECRHS Thorax 2002 57 1028 1033 10.1136/thorax.57.12.1028 12454296 PMC1758811

[B5] Litonjua AA Sparrow D Celedon JC DeMolles D Weiss ST Association of body mass index with the development of methacholine airway hyperresponsiveness in men: the Normative Aging Study Thorax 2002 57 581 585 10.1136/thorax.57.7.581 12096199 PMC1746377

[B6] Celedon JC Palmer LJ Litonjua AA Weiss ST Wang B Fang Z Xu X Body mass index and asthma in adults in families of subjects with asthma in Anqing, China Am J Respir Crit Care Med 2001 164 1835 1840 11734432 10.1164/ajrccm.164.10.2105033

[B7] Tantisira KG Litonjua AA Weiss ST Fuhlbrigge AL, for the Childhood Asthma Management Program Research Group: Association of body mass with pulmonary function in the Childhood Asthma Management Program (CAMP) Thorax 2003 58 1036 1041 10.1136/thorax.58.12.1036 14645968 PMC1746552

[B8] Schachter LM Salome CM Peat JK Woolcock AJ Obesity is a risk for asthma and wheeze but not airway hyperresponsiveness Thorax 2001 56 4 8 10.1136/thorax.56.1.4 11120896 PMC1745919

[B9] Bustos P Amigo H Oyarzun M Rona RJ Is there a causal relation between obesity and asthma? Evidence from Chile Int J Obes Relat Metab Disord 2005 29 804 809 10.1038/sj.ijo.0802958 15824747

[B10] Nicolacakis K Skowronski ME Coreno AJ West E Nader NZ Smith RL McFadden ER Jr Observations on the physiological interactions between obesity and asthma J Appl Physiol 2008 105 1533 1541 10.1152/japplphysiol.01260.2007 18787093 PMC2584837

[B11] Salome CM Munoz PA Berend N Thorpe CW Schachter LM King GG Effect of obesity on breathlessness and airway responsiveness to methacholine in non-asthmatic subjects Int J Obes (Lond) 2008 32 502 509 10.1038/sj.ijo.0803752 17955030

[B12] Scott HA Gibson PG Garg ML Wood LG Airway inflammation is augmented by obesity and fatty acids in asthma Eur Respir J 2011 38 594 602 10.1183/09031936.00139810 21310876

[B13] Scott HA Gibson PG Garg ML Pretto JJ Morgan PJ Callister R Wood LG Relationship between body composition, inflammation and lung function in overweight and obese asthma Respir Res 2012 13 10 10.1186/1465-9921-13-10 22296721 PMC3329414

[B14] Currie GP Fowler SJ Lipworth BJ Dose response of inhaled corticosteroids on bronchial hyperresponsiveness: a meta-analysis Ann Allergy Asthma Immunol 2003 90 194 198 10.1016/S1081-1206(10)62140-0 12602665

[B15] Ulrik CS Diamant Z Add-onmontelukast to inhaled corticosteroids protects against excessive airway narrowing Clin Exp Allergy 2010 40 576 581 20128823 10.1111/j.1365-2222.2010.03447.x

[B16] American Thoracic Society Guidelines for metacholine and exercise challenge testing-1999 Am J Respir Crit Care Med 2000 161 309 329 10619836 10.1164/ajrccm.161.1.ats11-99

[B17] Chai H Farr RS Froehlich LA Mathison DA MacLean JA Rosenthal RR Sheffer AL Spector SL Townley RG Standardization of bronchial inhalation challenge procedure J Allergy Clin Immunol 1975 56 323 327 10.1016/0091-6749(75)90107-4 1176724

[B18] Deesomchok A Fisher T Webb KA Ora J Lam Y Lougheed MD O’Donnel DE Effects of obesity on perceptual and mechanical responses to bronchoconstriction in asthma Am J Respir Crit Care Med 2010 181 125 133 10.1164/rccm.200906-0934OC 19910609

[B19] Holguin F Bleecker ER Busse WW Calhoun WJ Castro M Erzurum SC Fitzpatrick AM Gaston B Israel E Jariour NN Moore WC Peters SP Yonas M Teague G Wenzal SE Obesity and asthma: an association modified by age of asthma onset J Allergy Clin Immunol 2011 127 1486 1493 10.1016/j.jaci.2011.03.036 21624618 PMC3128802

[B20] Kwon JW Kim SH Kim TB Kim SH Park HW Chang YS Jang AS Cho YS Nahm DH Park JW Yoon HJ Cho YJ Choi BW Moon HB Cho SH Airway hyperresponsiveness is negatively associated with obesity or overweight status in patients with asthma Int Arch Allergy Asthma 2012 159 187 193 10.1159/000335926 22678356

[B21] Camargo CA Jr Weiss ST, Zhang S, Willett WC, Speizer FE: Prospective study of mass index, weight change, and risk of adult-onset asthma in women Arch Intern Med 1999 159 2582 2588 10.1001/archinte.159.21.2582 10573048

[B22] Chen Y Dales R Krewski D Breithhaupt K Increased effects of smoking and obesity on asthma among female Canadians: the National Population Health Survey, 1994–1995 Am J Epidemiol 1999 150 255 262 10.1093/oxfordjournals.aje.a009996 10430229

[B23] Castro-Rodriguez JA Holberg CJ Morgan WJ Wright AL Martinez FD Increased incidence of asthma like symptoms in girls who become overweight or obese during the school years Am J Respir Crit Care Med 2001 163 1344 1349 11371399 10.1164/ajrccm.163.6.2006140

[B24] Varraso R Siroux V Maccario J Pin I Kauffmann F Asthma severity is associated with body mass index and early menarche in women Am J Respir Crit Care Med 2005 171 334 339 10.1164/rccm.200405-674OC 15557134

[B25] Hamano N Terada N Maesako K Numata T Konno A Effect of sex hormones on eosinophilic inflammation in nasal mucosa Allergy Asthma Proc 1998 19 263 269 10.2500/108854198778557773 9801739

[B26] Hellings PW Vandekerckhove P Claeys R Billen J Kasran A Ceuppens JL Progesterone increases airway eosinophilia and hyperresponsiveness in a murine model of allergic asthma Clin Exp Allergy 2003 33 1457 1463 10.1046/j.1365-2222.2003.01743.x 14519155

[B27] Kim KM Kim SS Kwon JW Junq JW Kim TW Lee SH Min KU Kim YY Cho SH Association between subcutaneous abdominal fat and airway hyperresponsiveness Allergy Asthma Proc 2011 32 68 73 10.2500/aap.2011.32.3407 21262101

[B28] Van Harmelen V Reynisdottir S Eriksson P Thörne A Hoffstedt J Lönnqvist F Arner P Leptin secretion from subcutaneous and visceral adipose tissue in women Diabetes 1998 47 913 917 10.2337/diabetes.47.6.913 9604868

[B29] Sood A Ford ES Camargo CA Association between leptin and asthma in adults Thorax 2006 61 300 305 10.1136/thx.2004.031468 16540481 PMC2104595

[B30] Zarkesh-Esfafani H Pockley AG Wu Z Hellewell PG Weetman AP Ross RJ Leptin indirectly activates human neutrophils via induction of TNF-α J Immunol 2004 172 1809 1814 14734764 10.4049/jimmunol.172.3.1809

[B31] Chambers EC Heshka S Huffaker LY Xiong Y Wang J Eden E Gallagher D Pi-Sunyer FX Truncal adiposity and lung function in older black women Lung 2008 186 13 17 10.1007/s00408-007-9043-9 17952506

[B32] Sutherland TJT Goulding A Grant AM Cowan JO Williamson A Williams SM Skinner MA Taylor DR The effect of adiposity measured by dual-energy X-ray adsorptiometry on lung function Eur Respir J 2008 32 85 91 10.1183/09031936.00112407 18353855

[B33] Busse WW The relationship of airway hyperresponsiveness and airway inflammation: airway hyperresponsiveness in asthma: its measurement and clinical significance Chest 2010 138 4S 10S 10.1378/chest.10-0100 20668012 PMC2914527

[B34] Dixon AE Pratley RE Forgione PM Kaminsky DA Whittaker-Leclair LA Griffes LA Garudathri J Raymond D Poynter ME Bunn JY Irvin CG Effects of obesity and bariatric surgery on airway hyperresponsiveness, asthma control and inflammation J Allergy Clin Immunol 2011 128 508 515 10.1016/j.jaci.2011.06.009 21782230 PMC3164923

[B35] Langley SJ Goldthorpe S Custovic A Woodcock A Relationship among pulmonary function, bronchial reactivity and exhaled nitric oxide in a large group of asthmatic patients Ann Allergy Asthma Immunol 2003 91 398 404 10.1016/S1081-1206(10)61688-2 14582820

[B36] Ciprandi G Tosca MA Capasso M Exhaled nitric oxide in children with allergic rhinitis and/or asthma: a relationship with bronchial hyperreactivity J Asthma 2010 47 1142 1147 10.3109/02770903.2010.527026 20950134

[B37] Vignola AM Chanez P Campbell AM Souques F Lebel B Enander I Bousquet J Airway inflammation in mild intermittent and in persistent asthma Am J Respir Crit Care Med 1998 157 403 409 9476850 10.1164/ajrccm.157.2.96-08040

